# Cytotoxic CD4+ T Cells Drive Multiple Sclerosis Progression

**DOI:** 10.3389/fimmu.2017.01160

**Published:** 2017-09-20

**Authors:** Liesbet M. Peeters, Marjan Vanheusden, Veerle Somers, Bart Van Wijmeersch, Piet Stinissen, Bieke Broux, Niels Hellings

**Affiliations:** ^1^School of Life Sciences, Biomedical Research Institute, Hasselt University, Transnationale Universiteit Limburg, Diepenbeek, Belgium

**Keywords:** multiple sclerosis, prognosis, multimodal evoked potentials, CD4+CD28null T cells, personalized medicine, prognostic risk factor

## Abstract

Multiple sclerosis (MS) is the leading cause of chronic neurological disability in young adults. The clinical disease course of MS varies greatly between individuals, with some patients progressing much more rapidly than others, making prognosis almost impossible. We previously discovered that cytotoxic CD4+ T cells (CD4+ CTL), identified by the loss of CD28, are able to migrate to sites of inflammation and that they contribute to tissue damage. Furthermore, in an animal model for MS, we showed that these cells are correlated with inflammation, demyelination, and disability. Therefore, we hypothesize that CD4+ CTL drive progression of MS and have prognostic value. To support this hypothesis, we investigated whether CD4+ CTL are correlated with worse clinical outcome and evaluated the prognostic value of these cells in MS. To this end, the percentage of CD4+CD28null T cells was measured in the blood of 176 patients with relapsing-remitting MS (=baseline). Multimodal evoked potentials (EP) combining information on motoric, visual, and somatosensoric EP, as well as Kurtzke expanded disability status scale (EDSS) were used as outcome measurements at baseline and after 3 and 5 years. The baseline CD4+CD28null T cell percentage is associated with EP (*P* = 0.003, *R*^2^ = 0.28), indicating a link between these cells and disease severity. In addition, the baseline CD4+CD28null T cell percentage has a prognostic value since it is associated with EP after 3 years (*P* = 0.005, *R*^2^ = 0.29) and with EP and EDSS after 5 years (*P* = 0.008, *R*^2^ = 0.42 and *P* = 0.003, *R*^2^ = 0.27). To the best of our knowledge, this study provides the first direct link between the presence of CD4+ CTL and MS disease severity, as well as its prognostic value. Therefore, we further elaborate on two important research perspectives: 1° investigating strategies to block or reverse pathways in the formation of these cells resulting in new treatments that slow down MS disease progression, 2° including immunophenotyping in prediction modeling studies to aim for personalized medicine.

## Introduction

Multiple sclerosis (MS) is a chronic autoimmune disease of the central nervous system (CNS) characterized by inflammation, demyelination, and axonal loss (1). The leading hypothesis states that peripheral autoreactive T cells are activated after encountering self-antigens, after which they migrate across the blood–brain barrier into the CNS (2). Here, these T cells are re-activated, leading to a perpetuation of the local immune response, causing migration of other immune cells to the CNS and ultimately triggering tissue damage (3).

We hypothesize that cytotoxic CD4+ T cells (CTL) drive progression of MS and have prognostic value. CD4+ CTLs are increased in inflammatory conditions and while they protect against infections and tumors, they contribute to progression of autoimmunity and cardiovascular disease (Table 1). Here, we use the loss of the costimulatory molecule CD28 as a hallmark to define cytotoxic CD4+ T cells (CTLs), since this phenotype is very well described in various pathologies. Normally, CD28 costimulation is necessary for the activation, proliferation, and differentiation of T cells. In contrast, CD4+CD28null T cells are costimulation-independent and are still able to participate in immune responses (4). Chronic antigen stimulation leads to the gradual loss of CD28 on CD4+ T cells and induces several phenotypic and functional changes. Expanded CD4+CD28null T cells are found in about 20% of both MS patients as well as healthy controls without obvious differences in numbers (5). CD4+CD28null T cells have shorter telomeres, limited TCR diversity, increased resistance to apoptosis and expression of natural killer (NK) receptors. Furthermore, they produce large amounts of IFN-γ and cytotoxic molecules such as granzyme B and perforin (6).

**Table 1:** Involvement of CD4+ CTLs in different diseases.

Disease	CD4+ CTL markers	Evidence for CD4+ CTL disease association	Reference
**Infections**			
Persistent infections (EBV, CMV, HIV, parvovirus B19)	↑IFN-γ, IL-17, ↑↓, CD8, GrB, perforin, natural killer (NK) receptors, Eomes, T-bet	Control of infection: lyse infected cells *via* FasL and perforin mediated mechanisms	(7–13)
	↓CD28		
Acute infections (Influenza, Hantaan, and dengue virus)	↑T-bet, Blimp-1, GrB, perforin, CRTAM, Eomes, IFN-γ, CX3CR1, CD8α, NK receptors	Virus-specific cytotoxicity: directly kill infected MHC class II-expressing cells in an antigen-specific manner	(14–17)
	↓CD28		

**Tumors**	↑IFN-γ, GrB	Tumor cell lysis, tumor rejection/regression *via* MHC class-II-restricted antigen recognition	(18, 19)

**Autoimmunity**			
Crohn’s disease (IBD)	↑Eomes, Runx3, NK receptors, IL-17, IFN-γ, GrB, perforin	Increased in lamina propria, pro-inflammatory cytokine production by Th17- and Th1-like CTLs	(20–22)
Intestinal colitis (IBD)	↑Runx3, Eomes, CD8α, GrB, IFN-γ, T-bet, CRTAM	Enriched in colonic inflammatory sites, induce inflammation *via* CTL activity and cytokine production	(15, 21, 23, 24)
	↓ThPOK		
Multiple sclerosis (MS)	↑Eomes, GrB, perforin, IFN-γ, IL-17, T-bet, NK receptors, CD8, CX3CR1	Present in MS lesions and CSF, associate with neuroinflammation, kill oligodendrocytes, autoreactive and pro-inflammatory	(5, 6, 13, 25–28)
	↓CD28		
Rheumatoid arthritis	↑NK receptors, IL-17, IFN-γ, CD8, CX3CR1	Enriched at site of inflammation, autoreactive, cytotoxic and pro-inflammatory, correlate with severity and extra-articular manifestations	(6, 13, 29–31)
	↓CD28		
Autoimmune myopathies	↑IFN-γ, perforin, NK receptors	Pro-inflammatory, cytotoxic and tissue infiltrating	(5, 31–33)
	↓CD28		

**Cardiovascular diseases**			
Acute coronary syndrome (unstable Angina)	↑IFN-γ, perforin, GrB, NK receptors	Pro-inflammatory, lyse endothelial and vascular smooth muscle cells, autoreactive, correlate with recurrent events and poor outcome	(5, 31, 34)
	↓CD28		
Atherosclerosis	↑IFN-γ, perforin, GrB, NK receptors	Accumulate in rupture-prone regions, cytotoxic, augment apoptosis and necrosis	(34, 35)
	↓CD28		

*EBV, Epstein–Barr virus; CMV, cytomegalovirus; HIV, human immunodeficiency virus; IBD, inflammatory bowel disease; GrB, granzyme B*.

To determine the correlation of CD4+CD28null T cells with MS progression, we first assessed whether the presence of these cells is associated with higher disease severity using Kurtzke expanded disability status scale (EDSS) and multimodal evoked potentials (EP) as outcome measures. Second, to identify their prognostic value, we investigated the correlation between the baseline CD4+CD28null T cell percentage and the aforementioned outcome measures 3 and 5 years after baseline assessment.

## CD4+CD28null T Cells Drive MS Progression

Expansion of CD4+CD28null T cells is found in several autoimmune and chronic inflammatory diseases, including atherosclerosis (36), RA (37) Graves’ disease (38), Wegener’s granulomatosis (39) and sporadic inclusion body myositis (33) (Table 1). Importantly, the presence of these cells has been shown to be associated with disease severity and poor prognosis in atherosclerosis and RA (40). Recently, we showed that CD4+CD28null T cells are found in the CSF (unpublished data) and brain lesions of MS patients and migrate toward the inflamed CNS (5, 41) and correlate with disability in experimental autoimmune encephalomyelitis (EAE), an animal model of MS (42). While a direct link with MS disease severity was not demonstrated so far, indirect evidence, such as their target tissue infiltrating capacity and cytotoxic activity toward oligodendrocytes, certainly alludes to this (5, 26).

### The Presence of CD4+CD28null T Cells is Associated with Disease Severity

**Figure 1:**
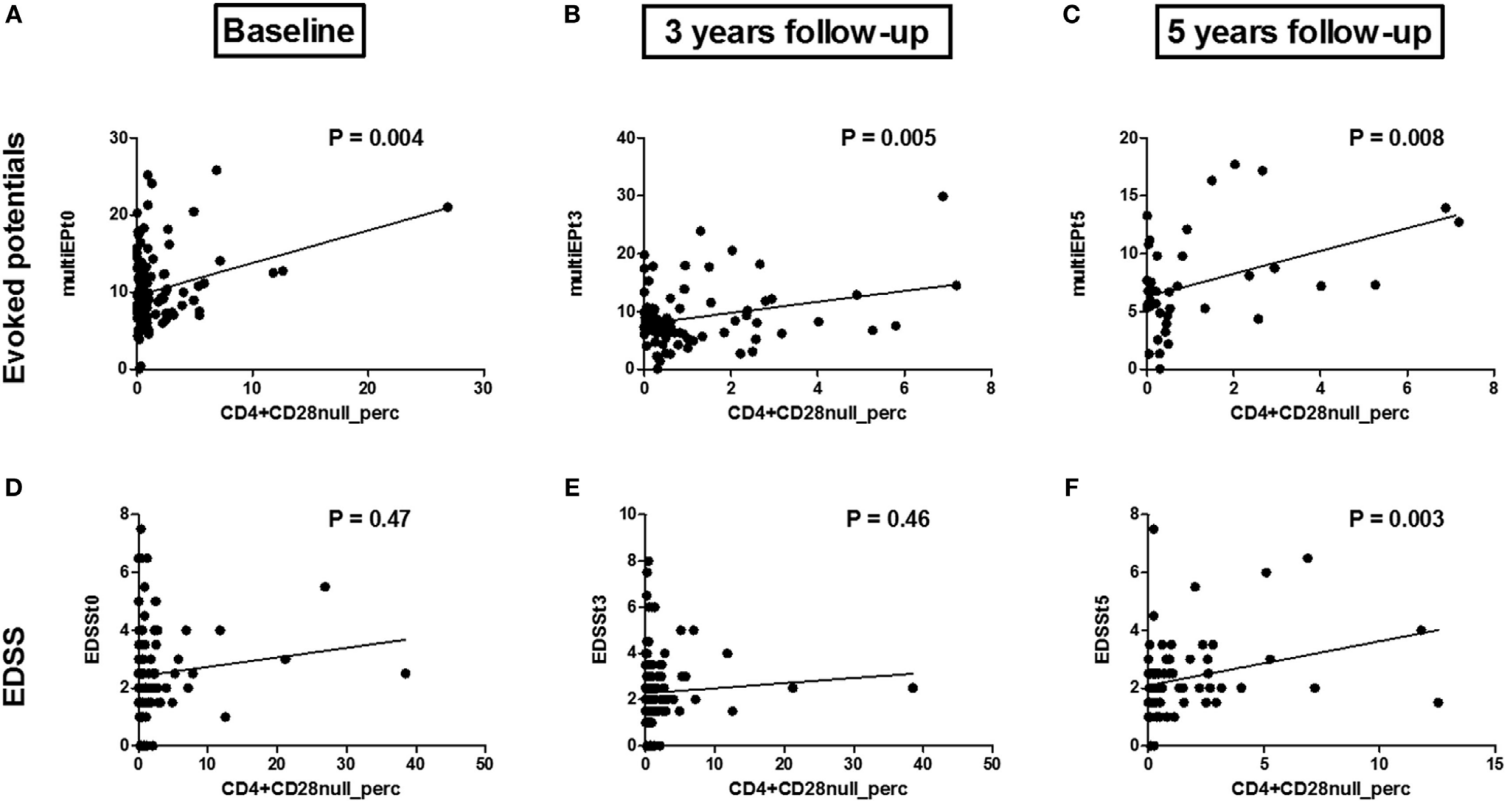
Associations between the CD4+CD28null T cell percentage and multi evoked potential (multiEP) and expanded disability status scale (EDSS): Association between the CD4+CD28null T cell percentage at baseline [**(A)**, *t*0, *N* = 109], 3 years [**(B)**, *t*3, *N* = 82], and 5 years [**(C)**, *t*5, *N* = 39] with multimodal evoked potentials (multiEP). Association between the CD4+CD28null T cell percentage and EDSS at baseline [**(D)**, *t*0, *N* = 121], EDSS 3 years post sampling [**(E)**, *t*3, *N* = 122], and 5 years post sampling [**(F)**, *t*5, *N* = 110]. *P*-values are corrected for disease duration and gender.

We investigated whether the CD4+CD28null T cell percentage is associated with a worse clinical disease course in 176 patients with relapsing-remitting MS (RRMS). Two clinical outcomes were measured: EDSS and multimodal EP combining information on motoric, visual, and somatosensoric EP. Baseline CD4+CD28null T cell percentage is associated with multimodal EP (*P* = 0.004, *R*^2^ = 0.28, Figure 1A), but not with EDSS (*P* = 0.47, *R*^2^ = 0.10, Figure 1D). EP are a more sensitive clinical outcome measure compared to EDSS. EP permit a quantitative assessment of the function of a few well-defined pathways of the CNS, reveal early infra-clinical lesions on the long sensory–motor pathways and have a predictive value regarding the evolution of disability (43–45). Indeed, several research groups have shown that multimodal EP allow monitoring of MS disability, both in cross-sectional and longitudinal studies (43, 44, 46, 47). EDSS and conventional magnetic resonance imaging (MRI) are the most widely used measures of disability. However, the potential limitations of these measures have received increasing attention. Indeed, EDSS appears to be insufficiently sensitive to robust changes in disability over short time frames and its application depends on the interpretation of the neurologist who performs the neurological examination. MRI shows of poor correlation with disability progression (48) and limited predictive value beyond the time of confirmation of diagnosis (49).

To the best of our knowledge, this is the first time a direct link between CD4+CD28null T cells and MS disease severity has been described. Other researchers have found indirect indications: NKG2D+CD4+ T cells were suggested to be associated with disease activity, since they are increased in the CSF of RRMS patients, with even higher frequencies in the active phases of MS (25). Furthermore, cytotoxic NKG2C+CD4+ T cells are more abundantly present in the blood and brain of MS patients (26). Eomes+CD4+ T cells were also increased in the peripheral blood and CSF of MS patients in the progressive phase of the disease (28). Perforin producing CD4+ T cells are activated and increased in active MS disease, again suggesting that CD4+ CTL may play a role in MS pathogenesis (50). Other CD4+ CTLs, namely cytolytic CD4+ T lymphocyte precursors and NKT cells, were also increased in MS patients (51).

### Mechanism(s) of CD4+ CTL Driven MS Progression

We propose that CD4CD28null T cells migrate toward the CNS to exert their cytotoxic and pro-inflammatory functions, in line with our recent finding that CD4+granzyme B+ T cells are present in the spinal cord of EAE mice and their percentages correlate with the extent of demyelination (42). Further research is necessary to elucidate by which mechanism(s) CD4+CD28null T cells contribute to the inflammatory response in autoimmune diseases such as MS. The observed impact of these cells on MS disease severity could be explained by a combination of following mechanisms: (1) production of pro-inflammatory cytokines, thereby augmenting inflammation, (2) infiltration into the CNS, leading to destruction of target tissue and cells *via* their cytotoxic activity leading to axonal loss similar as described by CD8+ T cells (52), and (3) recruitment of other harmful immune cells (e.g., macrophages) that contribute to inflammation (53, 54). In addition, it remains unknown whether these cells affect regulatory T cells, or whether they contribute to the differentiation of pathogenic T cell subsets (e.g., Th1, Th17).

### Inhibiting the Formation of CD4+ CTL could slow down MS Disease Progression

**Figure 2:**
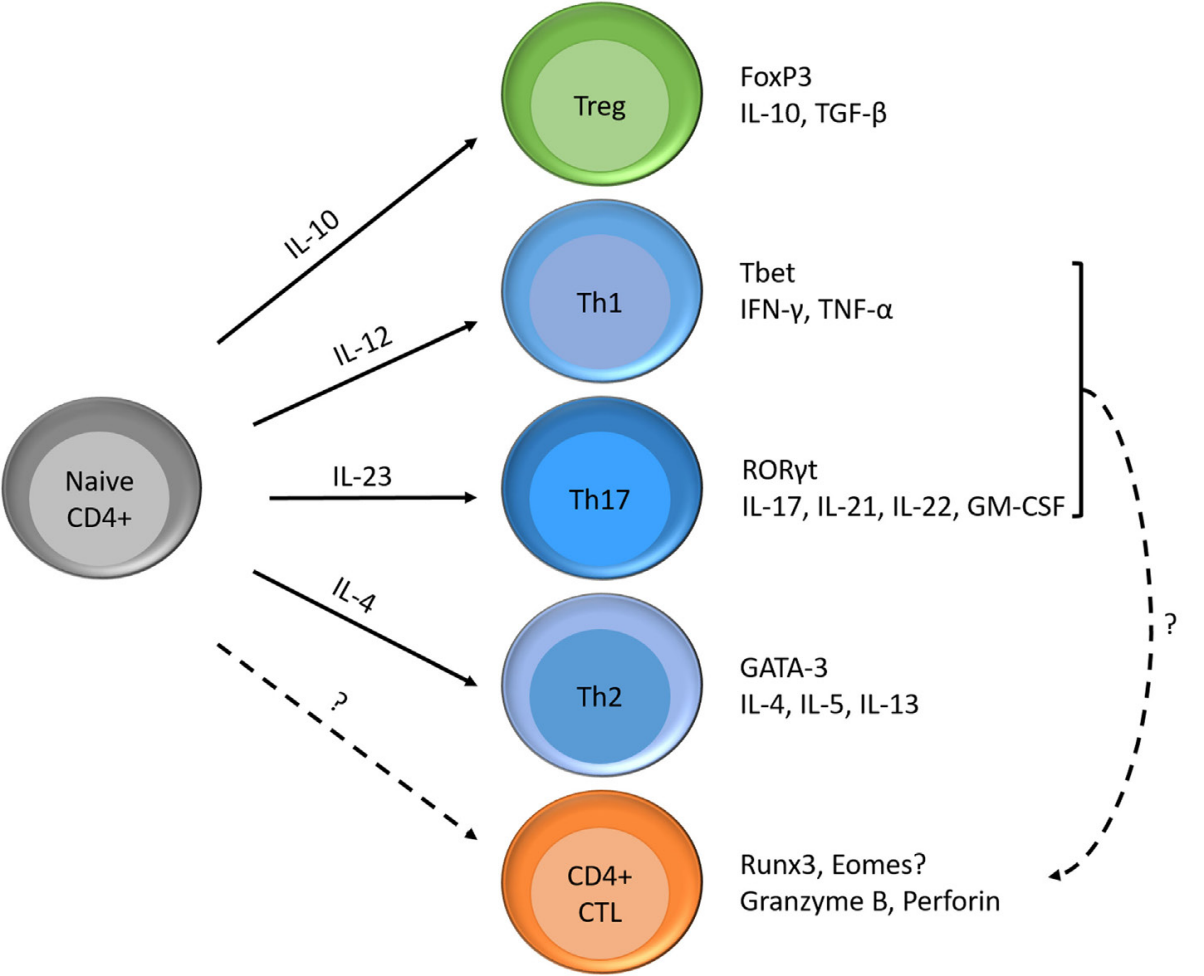
Differentiation possibilities of naïve CD4+ T cells and formation of CD4+ CTLs. Naïve CD4+ T cells can differentiate into T helper cells (e.g., Th1, Th2, and Th17), regulatory T cells and cytotoxic T cells according to the cytokines in their environment. Each subset has its specific transcription factor and produces its signature cytokines. Next to the differentiation of naïve T cells into CD4+ CTLs, Th1, or Th17 cells are also thought to become cytotoxic.

Investigating strategies to block or reverse pathways in the formation of these cytotoxic CD4 T cells may result in new treatments that slow down MS disease progression. Van Oosten et al. showed that intravenous treatment with an anti-CD4 monoclonal antibody was not able to reduce MS activity (55). However, they suggest that a more aggressive depletion of CD4+ cells might lead to a more substantial reduction in MRI activity. In addition, they did not investigate the presence of, or the effect of anti-CD4 antibodies on CD4+ CTL. CD4+CD28null T cells are only present in 20% of the general population (5) and are more resistant to apoptosis (6). Therefore, it is of interest to investigate the differentiation path from CD4+pre-CTLs to CD4+CD28null T cells, the probable end stage of CD4+ CTLs. CD4+ CTLs may originate either from naïve CD4+ T cells or through plasticity of CD4+ effector T helper cells (Figure 2). The formation of CD4+ CTLs is controlled by a combined action of transcriptional regulation and extracellular cues. These extracellular cues could consist of cytokines, T cell receptor stimulation and costimulatory signals, which lead to cytosolic signaling cascades (PI3K pathway and metabolic programs), ultimately altering gene transcriptional programs (17, 56). CD4+ CTL express part of the CD8 T cell lineage transcriptional program (10). Both CD4+ and CD8+ CTLs express, e.g., T-bet, Eomes, Runx3, and Hobit at higher levels compared to their naïve counterparts (57–60). Investigating the pathways involved in the formation of these cells could identify precursor cells defined by the expression of a certain transcription factor or phenotypic marker. Pre-CTLs may be used as an indicator for future expansion of CD4+CD28null T cells, making it more feasible to identify donors at risk of developing aberrant CD4+CTLs. Still, it should be kept in mind that these CD4+pre-CTLs still need to become cytotoxic, indicating that not all pre-CTLs lead to worse disease. Future studies are, therefore, warranted to provide clarity.

## CD4+ CTL have Prognostic Value and Should be Included in Personalized Prediction Models

The prognostic value of CD4+CD28null T cell percentage was tested by measuring EDSS and multimodal EP 3 and 5 years post sampling. After 3 years, there is a significant correlation between baseline CD4+CD28null T cells and multimodal EP (*P* = 0.005, *R*^2^ = 0.29, Figure 1B), but not with EDSS (*P* = 0.46, *R*^2^ = 0.06, Figure 1E). After 5 years, there is a significant correlation with both multimodal EP (*P* = 0.008, Figure 1C) and EDSS (*P* = 0.003, *R*^2^ = 0.27, Figure 1F). Several studies have shown the validity of multimodal EP in predicting disease severity in MS (43, 44, 61).

There is an urgent need for predictive tools that enable individualized prognostication and thus support personalized clinical decision-making. Considerable effort has been made to identify prognostic factors that estimate baseline risk, but the precision with which this can be done is still limited (62–64). For the era of personalized medicine to move forward in MS, the identification of personalized pathobiology will be critical to steer rational treatment selection (62). Decision support systems built on multidisciplinary data—clinical, laboratory, and genomic—may improve individualized prognostication and lead to better clinical management and decision-making. As shown for other diseases (65), phenotyping of immunological subsets could be valuable approach to help predict disease progression in MS.

## Conclusion

In conclusion, CD4+CD28null T cells are associated with MS disease severity and measuring circulating blood levels has prognostic value. It remains unclear to date how CD4+CD28null T cells arise, and at what stage CD4+ T cells acquire irreversible, full-blown cytotoxic activity that contributes to tissue damage and subsequent disease progression in MS. Finding the crucial factors that drive the process of CD4 CTL formation could feed the development of novel therapies to intervene with this process. Moreover, further research should focus on early identification of CD4+ CTL to identify these cells in more MS patients and earlier in the course of the disease thereby facilitating its use as prognostic factor.

## Ethics Statement

This study was approved by the ethical committee of the University Hasselt and informed consents were obtained from all donors.

## Author Contributions

LP analyzed the data and wrote the manuscript. MV determined the CD4+CD28null T cell percentages and contributed in writing the manuscript. VS, BW, PS, BB, and NH revised the manuscript, participated in the design, and cooperation of the project.

## Conflict of Interest Statement

The authors declare that the research was conducted in the absence of any commercial or financial relationships that could be construed as a potential conflict of interest.
